# Obesity and health behaviours of British adults with self-reported intellectual impairments: cross sectional survey

**DOI:** 10.1186/1471-2458-14-219

**Published:** 2014-03-03

**Authors:** Janet Robertson, Eric Emerson, Susannah Baines, Chris Hatton

**Affiliations:** 1 Centre for Disability Research, Lancaster University, Lancaster, UK; 2 Centre for Disability Research and Policy, University of Sydney, Sydney, Australia

## Abstract

**Background:**

People with intellectual disability have significantly higher age-adjusted rates of mortality and morbidity (including obesity) than their non-disabled peers. They are also significantly less likely to be physically active.

**Methods:**

Secondary analysis of de-identified cross-sectional data from the first two waves of *Understanding Society*, a new longitudinal study focusing on the life experiences of UK citizens. Interviews were undertaken with 50,994 individuals aged 16 and over in Wave 1 and 54,585 in Wave 2. Of these, 520 participants age 16–49 (1.8% of the unweighted age-restricted sample) were identified at either Wave 1 or Wave 2 as having self-reported intellectual impairments.

**Results:**

British adults with self-reported intellectual impairments have higher rates of obesity, inactivity, tobacco and alcohol use and poorer nutrition than their non-disabled peers. Adjusting risk estimates for between group differences in age, gender and exposure to material hardship indicated that a significant proportion of their increased risk of obesity, tobacco use and poorer nutrition may be attributable to their poorer living conditions (rather than their self-reported intellectual impairments per se).

**Conclusions:**

People with intellectual disabilities should begin to be regarded as a ‘vulnerable’ group in the context of public health policy and practice.

## Background

Intellectual disability refers to a significant general impairment in intellectual functioning that is acquired during childhood [1]. While estimates of the prevalence of intellectual disability vary widely, it has been estimated that approximately 2% of the adult population have intellectual disability [2,3]. People with intellectual disability have significantly higher age-adjusted rates of mortality and morbidity than their non-disabled peers [1,4,5]. This evidence, when combined with exposés of failings in healthcare systems [5-8] and increased attention to the human rights of disabled people [9], has led regulatory bodies and governments to stress the importance of reducing the health inequalities experienced by people with intellectual disability [10-14].

Health inequalities among adults with intellectual disability are particularly stark in relation to obesity and physical activity levels. There is now substantial evidence to suggest that people with intellectual disabilities are much more likely to be overweight or obese than the general population [15-28]. Increased risk of obesity among people with intellectual disabilities is associated with female gender, Down’s syndrome, less severe intellectual disability, living in less restrictive care environments and living in more deprived neighbourhoods [17,18,20,21,27,29,30]. Over 80% of adults with intellectual disabilities engage in levels of physical activity below the minimum recommended level [16-18,28,31,32], with people with more severe intellectual disabilities and people living in more restrictive care environments being at increased risk of inactivity [17].

Less is known about the prevalence of other pertinent health behaviours in people with intellectual disabilities. Studies in the UK and Australia have reported low intake of fruit and vegetables among adults with intellectual disability [17,28]. Fewer adults with intellectual disabilities who use intellectual disability services smoke tobacco or drink alcohol compared to the general population [17,22,28,33]. However, rates of smoking are considerably higher among adolescents with mild intellectual disabilities [34], parents with intellectual disability [35] and among people with intellectual disabilities who do not use intellectual disability services [36].

Current knowledge indicates that the reasons for the poorer health of people with intellectual disability primarily fall within two broad spheres. First, a range of secondary health conditions are associated with some specific causes of intellectual disability (e.g., higher rates of congenital heart defects in children with Down syndrome). Second, people with intellectual disability are much more likely than their non-disabled peers to be exposed to a range of well-established social determinants of poorer health (e.g., poverty, social exclusion, discrimination, reduced access to timely and effective healthcare) [1].

The aims of the present paper are: (1) to describe rates of obesity and pertinent health behaviours in British adults with and without intellectual disability; and (2) to estimate the extent to which any between-group differences in health status may reflect between-group differences in rates of exposure to socio-economic disadvantage.

## Methods

We undertook secondary analysis of de-identified cross-sectional data from the first two waves of *Understanding Society*[37]. Understanding Society is designed and conducted in accordance with the ESRC Research Ethics Framework and the ISER Code of Ethics. The University of Essex Ethics Committee approved Waves 1–5 of Understanding Society. Approval from the National Research Ethics Service was obtained for the collection of biosocial data by trained nurses in Waves 2 and 3 of the main survey (Understanding Society – UK Household Longitudinal Study: A Biosocial Component, Oxfordshire A REC, Reference: 10/H0604/2). The research presented here was based on secondary analysis of anonymised data sets that are available from the UK Data Archive. As such, further ethical approval was not required. Data were downloaded from the UK Data Archive (http://www.data-archive.ac.uk/). Full details of the surveys’ development and methodology are available in a series of reports [37-44], key aspects of which are summarised below.

### Samples

*Understanding Society* is a new longitudinal study focusing on the life experiences of UK citizens. In the first wave of data collection (undertaken between January 2009 and December 2011), random sampling from the Postcode Address File in Great Britain and the Land and Property Services Agency list of domestic properties in Northern Ireland identified 55,684 eligible households. Interviews were completed with 50,994 individuals aged 16 or older from 30,117 households, giving a household response rate of 54% and an individual response rate within co-operating households of 86% [38,44]. At Wave 2 interviews were completed with 54,585 individuals aged 16 or older from 30,428 households, giving an individual response rate within co-operating households of 91% [44]. Follow-up response rate from Wave 1 to Wave 2 was 74.7% [44].

### Procedures

Data collection was primarily undertaken using Computer Assisted Personal Interviewing. In Wave 2 health data (including height and weight) were collected by nurses for a subsample of 15,591 members of the cohort.

### Measures

#### Self-reported intellectual impairments

*Understanding Society* does not include information on the formal diagnosis of intellectual disability. As a result, we identified adults with self-reported intellectual impairments on the basis of self-reported long-term impairment or disability associated with difficulties in learning or understanding coexisting with low self-reported educational attainment. Low self-reported educational attainment was used as a selection criterion for two reasons: (1) to exclude from the intellectual disability group respondents with specific learning difficulties such as dyslexia; (2) as evidence that the person’s self-reported difficulty may have originated in childhood. Due to historical changes in educational qualifications and attainment in the UK, we further restricted our analysis to the age range 16–49.

*Understanding Society* uses a two-stage process for identifying participants with impairments or disability. First all participants are asked ‘*Do you have any long*-*standing physical or mental impairment*, *illness or disability*? *By* ‘*long*-*standing*’ *I mean anything that has troubled you over a period of at least 12 months or that is likely to trouble you over a period of at least 12 months*.’ If participants respond in the affirmative they are then asked ‘*Does this*/*Do these health problem* (*s*) *or disability* (*ies*) *mean that you have substantial difficulties with any of these areas of your life*? *Please read out the numbers from the card next to the ones which apply to you*.’ One response option is ‘*Memory or ability to concentrate*, *learn or understand*’. We initially identified adults in *Understanding Society* with self-reported intellectual impairments on the basis of their positive response to this routed question. We then excluded from this group participants who did not meet the additional criteria of having a highest level of educational attainment lower than a General Certificate of Secondary Education or equivalent. Overall 22.5% of *Understanding Society* participants reported lower than this level of attainment. This process identified 520 participants age 16–49 (1.8% of the unweighted age-restricted sample) at Wave 1 or Wave 2 as having self-reported intellectual impairments. In the Wave 2 subsample for which nurse measurements were taken, 142 participants age 16–49 (2.0% of the unweighted age-restricted sample) were identified as having self-reported intellectual impairments.

#### Health behaviours

In Wave 1 self-reported weight and height was collected and from these data BMI was calculated and obesity determined as BMI > 29. At Wave 2, weight and height were measured by a visiting nurse for a subsample of the *Understanding Society* cohort. The interview undertaken at Wave 2 collected self-report information on nutrition (type of bread and milk typically eaten, frequency of eating fruit and vegetables), physical exercise (walking), smoking (ever and currently) and alcohol use (typical number of days on which the informant drinks).

#### Socio-economic disadvantage

Socio-economic disadvantage was measured by a hardship scale and a measure of self-assessed financial status. The hardship scale, which was only administered at Wave 1, contained eight items: ‘*Next we have some questions about the sorts of things that some families*/*people have but which many people have difficulty finding the money for. For each of the following things please tell me the number from the showcard which best explains whether you* (*and your family*/*partner*) *have it or not. Do you* (*and your family partner*) *have*… (*1*) *A holiday away from home for at least one week a year*, *whilst not staying with relatives at their home*? (*2*) *Friends or family around for a drink or meal at least once a month*? (*3*) *Two pairs of all weather shoes for all adult members of the family*? (*4*) *Enough money to keep your house in a decent state of repair*? (*5*) *Household contents insurance*? (*6*) *Enough money to make regular savings of* £*10 a month or more for rainy days or retirement*? (*7*) *Enough money to replace any worn out furniture*? (*8*) *Enough money to replace or repair major electrical goods such as a refrigerator or a washing machine*, *when broken*?’ The response options for each item were ‘*1 I*/*We have this*, *2 I*/*We would like to have this but cannot afford this at the moment*, *3 I*/*We do not want*/*need this at the moment*, *4 Does not apply*’. A hardship score (0–8) based on the number of items that could not be afforded was derived for each participant.

Self-assessed financial status was assessed at Waves 1 and 2 by a single item: ‘*How well would you say you yourself are managing financially these days*? *Would you say you are*… *1 Living comfortably*, *2 Doing alright*, *3 Just about getting by*, *4 Finding it quite difficult or 5 finding it very difficult*?’.

## Results

Risk of self-reported intellectual impairments was unrelated to gender, but was significantly related to socio-economic disadvantage (at Wave 1 0.7% among participants who could not afford 0–1 items, 2.2% among participants who could not afford 2–4 items, 5.4% among participants who could not afford 5–8 items; Chi-Sq = 421.5 (2), p < 0.001). The prevalence of obesity and health behaviours (for men and women separately and combined) was first summarised through simple descriptive statistics (percentages). Unadjusted estimates of risk associated with self-reported intellectual impairments (odds ratios) are presented followed by estimates adjusted (using multivariate logistic regression) for between-group differences in: (1) age and gender; and (2) age, gender, material hardship and self-assessed financial status. Results are presented in Table 1.

**Table 1:** Obesity and Health Behaviours of Adults with and without Intellectual Disability

	**Prevalence (intellectual disability)**	**Prevalence (no intellectual disability)**	**Unadjusted risk**	**Risk adjusted for age and gender**	**Risk adjusted for age, gender and material hardship**
**Obesity**					
W1 (self-reported height and weight): All	26.6%	16.7%	1.78***	1.54**	1.16
			(1.35-2.34)	(1.16-2.03)	(0.87-1.55)
Men	20.3%	16.9%	1.06	0.96	0.82
			(0.66-1.70)	(0.59-1.56)	(0.50-1.34)
Women	31.9%	16.5%	2.49***	2.08***	1.45*
			(1.76-3.53)	(1.46-2.95)	**(1.01**-**2.08)**
W2 (nurse measured height and weight): All	41.3%	26.3%	2.01***	1.82**	1.29
			(1.39-2.91)	(1.25-2.65)	(0.88-1.90)
Men	39.4%	26.0%	1.67	1.58	1.23
			(0.93-3.00)	(0.87-2.88)	(0.67-2.26)
Women	42.7%	26.6%	2.28**	2.05**	1.38
			(1.41-3.69)	(1.26-3.33)	(0.83-2.28)
**Nutrition (W2)**					
Usually uses whole milk: All	35.1%	17.8%	2.28***	2.50***	1.50**
			(1.79-2.90)	(1.96-3.19)	**(1.16**-**1.93)**
Men	36.8%	19.4%	2.16***	2.23***	**1.39**
			(1.51-3.08)	(1.56-3.18)	**(0.96**-**2.00)**
Women	33.8%	16.4%	2.39***	2.84***	1.66**
			(1.72-3.32)	(2.04-3.96)	**(1.17**-**2.34)**
Usually eats white bread: All	53.3%	37.5%	1.85***	2.11***	1.50**
			(1.48-2.32)	(1.68-2.66)	**(1.19**-**1.90)**
Men	58.7%	42.0%	1.64**	1.74***	**1.22**
			(1.17-2.31)	(1.23-2.45)	**(0.86**-**1.73)**
Women	48.9%	33.9%	2.05***	2.49***	1.80**
			(1.51-2.77)	(1.83-3.39)	**(1.31**-**2.46)**
Eats fruit less than 4 days a week: All	65.5%	41.5%	2.76***	3.04***	2.03***
			(2.17-3.50)	(2.39-3.87)	**(1.58**-**2.60)**
Men	66.8%	46.9%	2.10***	2.17***	1.44*
			(1.47-2.99)	(1.52-3.10)	**(1.00**-**2.08)**
Women	64.4%	37.2%	3.45***	3.98***	2.67***
			(2.50-4.76)	(2.87-5.51)	**(1.91**-**3.73)**
Eats vegetables less than 4 days a week: All	48.5%	26.7%	2.81***	3.20***	2.19***
			(2.24-3.53)	(2.54-4.03)	**(1.73**-**2.78)**
Men	48.1%	29.8%	2.13***	2.24***	1.53*
			(1.52-3.00)	(1.59-3.16)	**(1.08**-**2.17)**
Women	48.9%	24.2%	3.54***	4.35***	3.01***
			(2.61-4.80)	(3.19-5.93)	**(2.19**-**4.13)**
**Physical activity (W2)**					
Not walked 10 min + in last 4 weeks: All	25.3%	10.7%	2.95***	2.78***	2.26***
			(2.28-3.82)	(2.14-3.61)	(1.73-2.95)
Men	22.2%	11.0%	2.49***	2.43***	1.90**
			(1.67-3.71)	(1.62-3.63)	(1.26-2.87)
Women	28.0%	10.3%	3.37***	3.08***	2.56***
			(2.40-4.74)	(2.18-4.34)	(1.82-3.66)
**Smoking (W2)**					
Ever smoked: All	76.2%	56.0%	2.73***	2.64***	1.90***
			(2.08-3.58)	(2.01-3.47)	**(1.44**-**2.51)**
Men	77.2%	58.3%	2.66***	2.62***	1.92**
			(1.76-4.03)	(1.73-3.97)	(1.26-2.92)
Women	75.3%	54.2%	2.78***	2.69***	1.91**
			(1.94-3.99)	(1.87-3.85)	(1.33-2.77)
Current smoker: All	55.7%	26.2%	3.44***	3.55***	2.03***
			(2.74-4.32)	(2.82-4.46)	**(1.59**-**2.58)**
Men	56.0%	28.1%	3.21***	3.23***	1.83**
			(2.28-4.51)	(2.30-4.54)	**(1.29**-**2.62)**
Women	55.5%	24.7%	3.65***	3.88***	2.24***
			(2.69-4.96)	(2.85-5.28)	**(1.62**-**3.10)**
**Alcohol use (W2)**					
Drinks daily: All	9.4%	4.7%	2.41***	1.96**	2.01**
			(1.57-3.69)	(1.27-3.03)	(1.29-3.13)
Men	14.5%	6.4%	2.92**	2.53**	2.36**
			(1.71-4.98)	(1.47-4.36)	(1.36-4.13)
Women	5.0%	3.4%	1.80	1.30	1.49
			(0.86-3.79)	(0.61-2.75)	(0.70-3.21)

Notes: *** p < 0.001, ** p < 0.01, * p < 0.05.

Risk estimated in bold indicates a significant reduction in risk from the previous estimate after adjustment for material hardship (point adjusted estimate lies outside 95% CI for previous estimate).

As can be seen, unadjusted risk for obesity and all indicators of less healthy behaviours was significantly greater among adults with self-reported intellectual impairments (risk range 1.78 (Wave 1 obesity) to 3.44 (current smoker)). Unadjusted risk for obesity and indicators of less healthy behaviours was significantly greater for both men and women with self-reported intellectual impairments with just two exceptions; obesity was not significantly greater for men, alcohol consumption was not significantly greater for women. Adjusting risk estimates to take account of between-group differences in age and gender had little impact. Further adjusting risk estimates to take account of between-group differences in exposure to material hardship and self-assessed financial status reduced the risk associated with self-reported intellectual impairments for obesity and all indicators of less healthy behaviours with one exception, alcohol use. For 17 of the 30 analyses (57%) this adjustment resulted in statistically significant reductions in risk associated with self-reported intellectual impairments. These effects were most marked in relation to nutrition and smoking.

While obesity rates based on nurse measures were markedly higher than obesity rates based one year previously on self-reported height and weight, there was a strong association between the two measures with 90% of people identified as obese at Wave 1 also being identified as obese at Wave 2 (Kappa = 0.67; OR = 69.9 (57.5-85.1), p < 0.001).

## Discussion

Our results indicate that: (1) British adults with self-reported intellectual impairments have higher rates of obesity, inactivity, tobacco and alcohol use and poorer nutrition than their non-disabled peers; and (2) that a significant proportion of their increased risk of obesity, tobacco use and poorer nutrition may be attributable to their poorer living conditions (rather than their self-reported intellectual impairments per se).

These results add to existing knowledge about the health inequalities faced by people with intellectual disability in four important ways. First, they are based on the analysis of contemporary population-based sampling frames, a relative rarity in this field of study [1].

Second, being based on samples drawn from general households participants are likely to include adults with less severe intellectual disability who may not be in receipt of specialised disability services. Given that most intellectual disability research is based on convenience samples drawn from the users of specialised disability services (typically people with more severe intellectual disability), very little is currently known about the health or well-being of this group that has been termed the ‘hidden majority’ of adults with intellectual disability [35,36,45]. The results of the present study are consistent with this sparse literature in indicating higher rates of alcohol and tobacco use among adults with intellectual disability.

Third, by adjusting risk estimates for between-group differences in exposure to material hardship and self-assessed financial status, the results add to the growing literature suggesting that a significant proportion of the health inequalities experienced by people with intellectual disabilities may be attributable to their poorer living conditions (rather than their intellectual disability per se) [1,46]. Thus, for example, the significantly elevated rates of obesity among women with intellectual disability were either significantly reduced or eliminated when adjusted for these indicators of socio-economic position, raising the question of whether the observed higher rates of obesity are related to intellectual disability per se or may be attributable to the higher rates of poverty experienced by women with intellectual disability.

Finally, the present study is one of the few in this field to stratify results by gender [1]. Important interactions between gender and intellectual disability emerged in three areas. Increased risk of obesity among adults with intellectual disability was largely specific to women, with a similar trend evident for inactivity. In contrast increased risk of frequent alcohol use among adults with intellectual disability was largely specific to men.

However, there are five limitations to the study that should be kept in mind when considering the salience and implications of these results. First, it is not possible to estimate the sensitivity or specificity of the method used to identify participants with potential intellectual disability. Hence our use of the term self-reported intellectual impairments. It should be noted, however, that: (1) the overall prevalence rates lies within the expected boundaries for intellectual disability [2,3]; (2) the prevalence of self-reported intellectual impairments varied with indicators of socio-economic position in a manner consistent with previous epidemiological research [3]. Second, the use of a general household sampling frame excludes people with (primarily more severe) intellectual disability living in institutional forms of residential care. Third, the consent and interview procedures used in *Understanding Society* are also likely to exclude people with more severe intellectual disability from participating. Consequently, the results are likely to be particularly relevant to understand the health of British adults with less severe intellectual disability. Fourth, whilst obesity was assessed by nurse measures at Wave 2, all other health behaviours were self-reported and it is not possible to assess the accuracy of such self-reports. Finally, while the cross-sectional analyses presented in this paper are consistent with the hypothesis that the poorer health of adults with intellectual disability may be partially attributable to their poorer living conditions, it is not possible to rule out other explanations (e.g., people with intellectual disability are more susceptible to downward social mobility if they have poor health than their non-disabled peers).

## Conclusions

British adults with self-reported intellectual impairments have higher rates of obesity, inactivity, tobacco and alcohol use and poorer nutrition than their non-disabled peers. A significant proportion of their increased risk of obesity, tobacco use and poorer nutrition may be attributable to their poorer living conditions (rather than their intellectual disability per se). Effectively addressing health inequalities requires policies that are sensitive to the particular personal and social contexts of ‘vulnerable’ groups [47]. People with intellectual disabilities should begin to be regarded as ‘vulnerable’ groups in the context of public health policy and practice. A similar case has been made for regarding people with disabilities in general as a ‘vulnerable group’ [46,48].

## Competing interests

The authors declare that they have no competing interests.

## Authors’ contributions

JR participated in the design of the study and coordinated the drafting of the manuscript. EE participated in the design of the study, performed the statistical analysis and helped to draft the manuscript. SB and CH participated in the design of the study. All authors read and approved the final manuscript.

## Pre-publication history

The pre-publication history for this paper can be accessed here:

http://www.biomedcentral.com/1471-2458/14/219/prepub
